# Study on pecan seed germination influenced by seed endocarp

**DOI:** 10.1515/biol-2022-0088

**Published:** 2022-08-10

**Authors:** Jia Liu, Tingting Xue, Liying Ren, Mingxia Cui, Tao Jiang, Xutao Yang

**Affiliations:** Department of Civil and Architecture and Engineering, Chuzhou University, Anhui, China; Department of Shiwaitaoyuan Ecoagriculture Cooperative, Anhui, China; Department of Chuzhou Forestry Station, Anhui, China

**Keywords:** pecan, seed germination, endocarp, chemical dormancy, mechanical dormancy

## Abstract

Nondeep physiological dormancy exists in freshly harvested pecan (*Carya illinoinensis*) seed, and the endocarp inhibits the seed germination. New methods were tried to detect if “chemical dormancy” or “mechanical dormancy” was caused by the endocarp. The germination of freshly harvested pecan seed with the removal of different parts of the endocarp and the fracture pressure of the endocarp of pecan seed soaked in water at different temperatures were tested. The results showed that (1) there was no significant difference in germination rate between the pecan kernel keeping in touch and out of touch with the same part of the endocarp, (2) whether a part of endocarp was removed to expose the radicle, preventing endocarp from splitting by glue inhibited the radicle elongation significantly, (3) the fracture pressure of the endocarp decreased significantly over water uptake time, and (4) little difference in the fracture pressure of the endocarp between different soaking temperatures. In conclusion, it suggested that (1) the endocarp caused “mechanical dormancy” but “chemical dormancy,” (2) the prevention of radicle elongation was due to the endocarp pressuring the cotyledon rather than the direct physical restriction on the radicle, and (3) the radicle elongation seemed to be able to respond to the suture split and the pressure on the cotyledon.

## 1 Introduction

Pecan (*Carya illinoinensis*) belongs to the family Juglandaceae and originates from North America. Pecan is an important economic tree with excellent fruit, oil, and wood. Pecan nurseries in China have developed rapidly since 2010. China imported a total of about 100 t of pecan seeds from 2010 to 2013, producing 150 million seedlings [1]. Freshly harvested pecan seeds germinate at a high temperature (30–35°C), which is not conducive to the intensive production of pecan seedlings, and by removing the endocarp, pecan seeds germinate more quickly, and the minimum temperature for germination decreases to 25°C [2]. It suggested that nondeep physiological dormancy exists in freshly harvested pecan seeds, and the endocarp inhibits seed germination.

According to Nikolaeva’s types of dormancy [3], seed shells can cause physical dormancy, “chemical dormancy,” and “mechanical dormancy.” Baskin and Baskin [4] suggest that “chemical dormancy” and “mechanical dormancy” are parts of physiological dormancy. Seed covering structures cause dormancy by a reduced rate of imbibition, restricting the movement of oxygen to the embryo, presence of inhibitors, and physical restriction [5].

Although pecan endocarp is hard, it can be permeable to water; thus, the pecan seed does not have physical dormancy. Scanning electron microscopy of pecan endocarp found that the endocarp consists of isodiametric sclereids, flat sclereids, and created oblatoid cork cells. Vascular bundles, ellipsoidal sclereids, and dumbbell- or kidney-shaped sclereids are found close to the suture [6].

Germination inhibitors in the fruit wall cause chemical dormancy [3]. However, the presence of chemical dormancy is hard to confirm. In lots of previous studies and a few recent studies [7] on chemical dormancy, extracts of seeds were treated on the same or other species and/or seed shells were removed. However, it is hard to determine if there are germination inhibitors or “chemical dormancy” because (1) removal of fruit wall releases mechanical restriction and removes inhibitors, and (2) tolerance of embryos to inhibitors may depend on PD [5]. In addition, leaching is also hard to confirm if there is a germination inhibitor in endocarp because germination inhibitors may exist in not only the endocarp but also the seed coat.

Our objective is to study the cause that the endocarp inhibits pecan seed germination.

## 2 Materials and methods

### 2.1 Materials

Pecan “Jinghua” seeds produced in Sihong County, Jiangsu Province, were supplied by the Jiangsu Academy of Forestry. Freshly harvested seeds were dried and stored in a dry and well-ventilated room.

### 2.2 Chemical dormancy test

**Figure 1:**
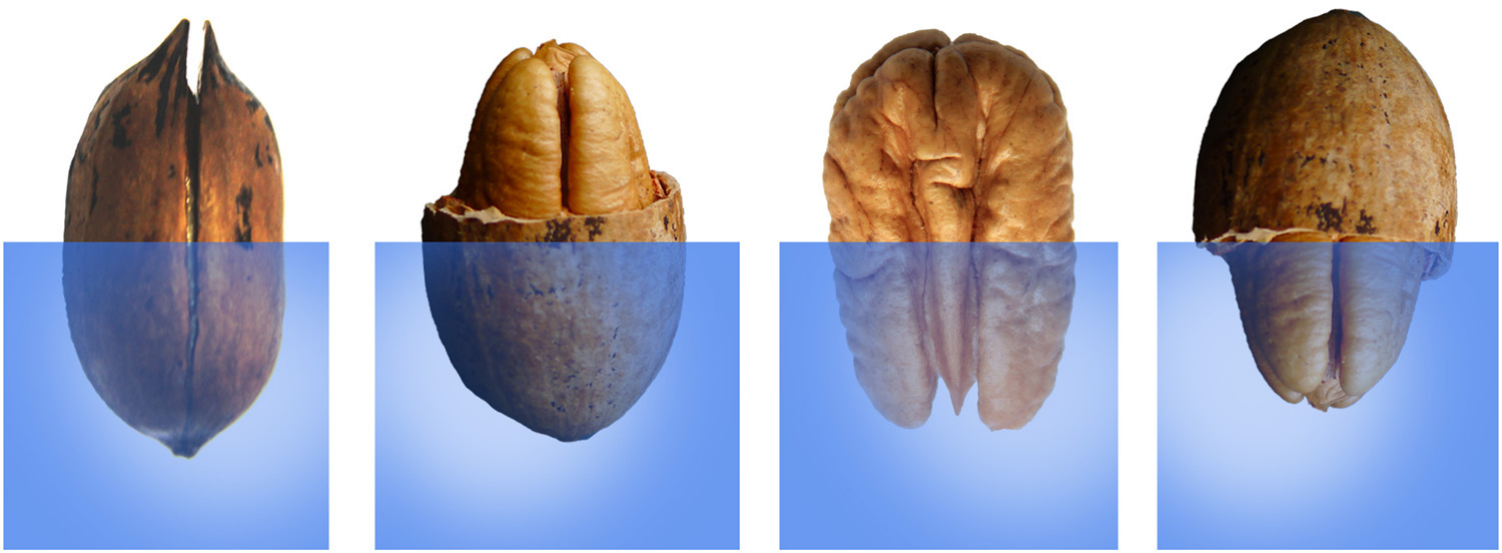
Representation of different parts of the endocarp removed for the germination test. (a) The half endocarp close to pecan radicle was broken along to the suture, and another half endocarp was exposed to water. (b) The half endocarp close to pecan radicle was removed, and another half endocarp was exposed to water. (c) Whole pecan endocarp was removed, and the half kernel with the radicle was exposed to water. (d) The half endocarp close to pecan radicle was removed, and the half kernel with radicle was exposed to water.

Treatment A: The half endocarp close to the pecan radicle was broken along with the suture, and another half endocarp was exposed to water in a small ice cube tray (size: 2.5 cm × 2.5 cm, height: 2 cm) (Figure 1a).

Treatment B: The half endocarp close to the pecan radicle was removed, and another half endocarp was exposed to water in a small ice cube tray (Figure 1b). The water level is lower than the half endocarp.

Treatment C: Whole pecan endocarp was removed, and the half kernel with the radicle was exposed to water in a small ice cube tray (Figure 1c).

Treatment D: The half endocarp close to the pecan radicle was removed, and the half kernel with the radicle was exposed to water in a small ice cube tray (Figure 1d).

Pecan seeds with four treatments were incubated at 25°C. Each treatment used four replications of 25 seeds. The seeds were kept moist with lids and distilled water on the trays whenever necessary. Germination of the seeds was recorded daily for 45 days.

### 2.3 Mechanical dormancy test

Seeds were randomly selected and followed different treatments:

Treatment 1: Control.

Treatment 2: The endocarp suture was reinforced by glue to prevent endocarp from splitting.

**Figure 2:**
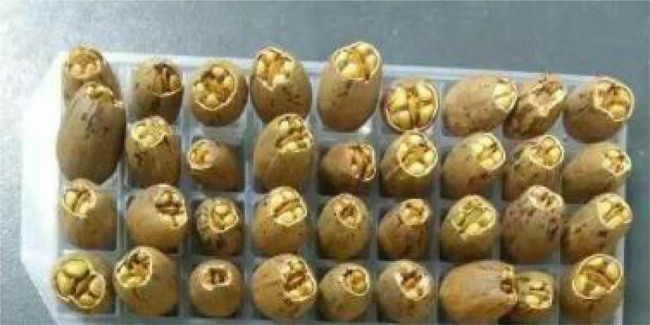
Removal of a small part of the endocarp around the radicle.

Treatment 3: A small part of the endocarp was removed to expose the radicle (Figure 2).

Treatment 4: A small part of the endocarp was removed to expose the radicle. Then, the endocarp suture was reinforced by glue to prevent the endocarp from splitting.

Pecan seeds with four treatments were incubated at 30°C. Each treatment used four replications of 25 seeds. The seeds were kept moist with lids and distilled water on the trays whenever necessary. Germination of the seeds was recorded weekly for 30 days. After 30 days, the ungerminated seed was dissected to check the elongation of the radicle.

### 2.4 Fracture pressure of the pecan endocarp test

Pecan seeds of similar size and shape were selected and soaked in water at 15, 25, and 30°C. The ungerminated seeds were collected for fracture pressure tests after 0, 5, 10, 15, and 25 days. Each treatment used 100 replications.

Electronic universal testing machine (SANS-CMT6104) was used to measure the fracture pressure of the endocarp. The measurement accuracy is 0.5%, the deformation measurement range is 0.02–800 mm, and the deformation resolution is 1/300,000 FS.

Each pecan seed was placed upright, and the individual sample was pressurized at 100 mm/min loading speed. The pressures were recorded when the endocarps were fractured. Furthermore, the locations of the fracture at the endocarps were recorded.

### 2.5 Data analysis

Statistical analyses of the data were performed using SPSS 20.0. The results were subjected to an analysis of variance to detect differences between the mean values and the mean values compared using the least significant difference (multiple comparisons).

## 3 Results

### 3.1 Chemical dormancy test

**Figure 3:**
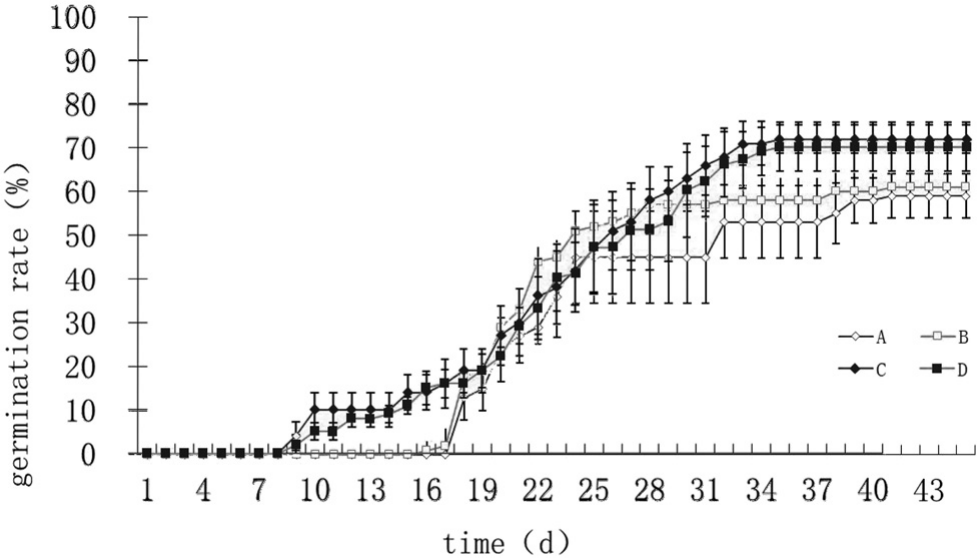
Germination of pecan seeds with four endocarp removing treatments at 25°C: A: The half endocarp close to the pecan radicle was broken along with the suture, and the radicle was exposed to air. B: The half endocarp close to pecan radicle was removed, and the radicle was exposed to air. C: Whole pecan endocarp was removed, and the radicle was exposed to water. D: The half endocarp close to pecan radicle was removed, and the radicle was exposed to water. Error bars indicate standard errors of the mean.

Figure 3 and Table 1 show the results of germination of pecan seeds with four treatments at 25°C. Seeds with treatments A and B were germinated from 16 to 38 days, and seed germination rates were 60 and 64%. There was no significant difference in germination rate between the pecan seeds with treatment A and treatment B. Seeds of treatments C and D were germinated from 9 to 30 days, and seed germination rates were 70 and 72%. There was no significant difference in germination rate between the pecan seeds with treatment C and treatment D.

**Table 1:** Germination rate and germination time of pecan seeds with four treatments at 25°C

Treatment	Germination rate (%)	Time (day)	
		*T*_*h*_ (day)	*T*_max_ (day)
A	60 ± 5^b^	22 ± 1^a^	38 ± 3^a^
B	64 ± 4^b^	21 ± 1^a^	37 ± 4^a^
C	72 ± 3^a^	23 ± 2^a^	33 ± 1^b^
D	70 ± 6^a^	23 ± 1^a^	33 ± 2^b^

Note: A: The half endocarp close to the pecan radicle was broken along with the suture, and the radicle was exposed to air. B: The half endocarp close to the pecan radicle was removed, and the radicle was exposed to air. C: Whole pecan endocarp was removed, and the radicle was exposed to water. D: The half endocarp close to pecan radicle was removed, and the radicle was exposed to water. *T*_*h*_: The time when the seed germination rate reaches half of the maximum germination rate; *T*_max_: The time when the seed germination rate reaches the maximum germination rate. Different letters in the same column of values show significant differences (Duncan’s multiple comparisons): *p* < 0.05.

Therefore, there was no significant difference in germination rate between the pecan kernel keeping in touch and out of touch with the same part of the endocarp.

The germination rates of the pecan seeds with treatment A and treatment B were significantly lower than the germination rates of the pecan seeds with treatment C and treatment D. The pecan seeds with treatment A and treatment B need more germination time than the pecan seeds with treatment A and treatment B.

### 3.2 Mechanical dormancy test

The seed germination rate of control (treatment 1) was 84 ± 3%. No seed germinated after the endocarp suture was reinforced with glue (treatment 2), and no radicle of the seeds elongated. After the small part of endocarp was removed (treatment 3), 62 ± 10% seeds germinated. After the small part of endocarp was removed and the suture was reinforced (treatment 4), 21 ± 8% seeds germinated. The differences in germination rate between the four treatments were significant.

The results showed whether a part of endocarp was removed to expose the radicle, preventing endocarp from splitting by glue inhibited the radicle elongation significantly. The inhibition of radicle elongation was not due to the direct physical restriction of the small part of endocarp close to the radicle.

### 3.3 Fracture pressure of the pecan endocarp test

Table 2 shows the fracture pressure of pecan endocarp during water uptake at different temperatures. The fracture pressure of the endocarp decreased significantly over water uptake time. The fracture pressure decreased from about 0.3 kN to about 0.2 kN. There is little difference in the fracture pressure of the endocarp between different soaking temperatures.

**Table 2:** Fracture pressure of pecan endocarp during water uptake at different temperatures

	Pressure that fractured seed endocarps/kN		
Soaking time	15℃	25℃	30℃
0 day (dry seed)	0.30 ± 0.15^ab^	0.30 ± 0.15^ab^	0.30 ± 0.15^ab^
5 day	0.33± 0.10^a^	0.28 ± 0.04^a^	0.27 ± 0.11^ab^
10 day	0.32 ± 0.06^a^	0.29 ± 0.10^a^	0.28 ± 0.12^ab^
15 d	0.27 ± 0.05^ab^	0.22 ± 0.08^b^	0.23 ± 0.05^b^
25 day	0.21 ± 0.08^b^	0.22 ± 0.12^b^	0.21 ± 0.06^b^

Different letters in the same column of values show significant differences (Duncan’s multiple comparisons): *p* < 0.05.

In addition, all the fractured endocarps did not crack along the suture, and the fracture locations in the endocarps were random.

## 4 Discussion

### 4.1 Chemical dormancy

Fruit wall removal releases mechanical restriction and inhibitors, so an increase in germination percentages by removing the fruit wall is hard to confirm the presence of chemical dormancy [5]. Few studies mentioned chemical dormancy in recent years. For example, the conclusion of ABA dose–response quantification of germination is that ABA is a key component of a pericarp-mediated chemical dormancy in *Lepidium draba* [8].

We tried to use a new method to detect if there is “chemical dormancy” caused by the endocarp. In our test, the mechanical restriction caused by half of endocarp was removed, and the germination of the pecan kernel keeping touch and out of touch with the same part of the endocarp was tested. No significant difference in germination rate between the pecan seeds with treatment A and treatment B (Figure 3) suggested that there is no germination inhibitor in the half of the endocarp. Similarly, no significant difference in germination rate between the pecan seeds with treatment C and treatment D (Figure 3) suggested that there is no germination inhibitor in the other half of endocarp. Therefore, there is no “chemical dormancy” caused by the endocarp.

In addition, the lower germination rates of the pecan seeds with treatment B compared to the pecan seeds with treatment D may be due to the lack of water in the exposed radicle of the pecan seeds with treatment B. The longer germination time of the pecan seeds with treatment B compared to the pecan seeds with treatment D may be due to the half of endocarp delayed water uptake of the pecan seeds with treatment B.

### 4.2 Mechanical dormancy

Before pecan seed germination, the endocarp split along the suture, but in the fracture pressure test, the location of the fracture was not the suture. Obviously, the crack was initiated at the weak locations. It suggested that the bonding force of the suture was released during germination. It indicated that the embryo growth potential of pecan was necessary for the suture split, but at 25°C, the embryo growth potential was not enough to overcome the physical restriction. It supported that mechanical dormancy is a part of physiological dormancy.

The results showed that reinforcing the suture with glue strongly inhibited pecan seed germination, even though a part of endocarp was removed to expose the radicle. It suggested that the prevention of radicle elongation was due to the endocarp pressuring the cotyledon rather than the direct physical restriction on the radicle. It is reasonable for the successful germination of some seeds with hard shells. It was reported that the fracture pressure of walnut, almond, and macadamia nut was 378 ± 165, 895 ± 181, and 2,364 ± 645 N, respectively [9]. It is hard to pierce through pecan endocarp by needle, letting alone the soft radicle. It indicated that the radicle elongation seemed to be able to respond to the suture split and the pressure on the cotyledon.

In pecan germination, endocarp split and radicle elongation are sequential events. A lot of species, such as *Lepidium* [10], *Trollius* [11], *Chenopodium* [12], *Nicotiana* [13], and *Petunia* [14], exhibit two-step germination, in which testa rupture and endosperm rupture are also sequential events. Therefore, the response of radicle to seed covering rupture may exist widely. This mechanism is unclear and needs more research.

## 5 Conclusions

1) The endocarp caused “mechanical dormancy” but “chemical dormancy.”
2) The prevention of radicle elongation was due to the endocarp pressuring the cotyledon rather than the direct physical restriction on the radicle.
3) The radicle elongation seemed to be able to respond to the suture split and the pressure on the cotyledon.
